# Infant Feeding Regimens and Gastrointestinal Tolerance: A Multicenter, Prospective, Observational Cohort Study in China

**DOI:** 10.1177/2333794X17750271

**Published:** 2018-01-09

**Authors:** Meng Mao, Lan Zhang, John Ge, Jian Yan, Robert Northington, Manjiang Yao, Joyce Nowacki, Nicholas P. Hays

**Affiliations:** 1 Chengdu Women’s and Children’s Central Hospital, Chengdu, Sichuan Province, China; 2 Wyeth Nutrition (China) Company Ltd, Shanghai, China; 3 Nestlé Nutrition, Vevey, Switzerland; 4 Nestlé Nutrition, King of Prussia, PA, USA

**Keywords:** infant formula, stool characteristics, gastrointestinal tolerance, breastfeeding, mixed feeding, observational study, China, Infant Gastrointestinal Symptom Questionnaire, oligofructose, sn-2 palmitate

## Abstract

To study feeding tolerance in infants fed formula with increased *sn*-2 palmitate and oligofructose (*sn*-2+OF) in a real-world setting, healthy Chinese infants were enrolled in this 48-day observational study on their current feeding regimens: exclusively breastfed (BF; n = 147), exclusively *sn*-2+OF formula-fed (FF; n = 150), or mixed-fed with breast milk and *sn*-2+OF formula (MF; n = 163). Throughout the study, incidence (90% confidence interval) of hard stools was ≤2.1% (0.0-5.3) in FF and 0.8% (0.0-3.5) in MF, with no hard stools in BF. Incidence of watery stools was ≤5.0% (1.0-9.2) in FF and ≥5.1% (2.4-9.3) in MF and BF. Gastrointestinal tolerance scores, although low in all groups (lower scores indicating better tolerance), were slightly higher (*P* ≥ .03) in FF (17.5 ± 4.8) and MF (18.2 ± 5.0) versus BF (16.3 ± 3.2) at mid-study; this difference disappeared at study end. Overall, low incidences of hard and watery stools and good feeding tolerance were observed in infants fed *sn*-2+OF formula.

## Introduction

Stool frequency and consistency often differ between breastfed and formula-fed infants. Formula-fed infants generally have firmer, less frequent stools compared with breastfed infants,^1,2^ likely due in part to differences in stool chemical composition, particularly in the lipid and mineral fractions.^3^ In addition, undesirable gastrointestinal (GI) effects, such as constipation, colic, flatulence, and regurgitation, are relatively common in young infants,^4,5^ closely dependent on the infant’s diet,^6,7^ and are associated with increased parental concerns regarding infant health. Breastfeeding is the gold standard of infant nutrition, but for those infants who must be formula-fed, it is important to match the stooling and GI outcomes of their breastfed peers as closely as possible.

As a means of addressing the issues of stool hardness and GI tolerance in formula-fed infants, alterations to both formula fat blends and carbohydrate composition have been useful.^8,9^ Formulas with fat blends that are compositionally and structurally more similar to breast milk than standard formulas have been developed containing triglycerides with an increased proportion of palmitic acid in the *sn*-2 position. During intestinal digestion of such formulas, a smaller amount of free palmitic acid is produced compared with formulas with standard vegetable oil fat blends. As a result, feeding formulas containing high *sn*-2 palmitate results in reduced formation and fecal excretion of insoluble calcium fatty acid soaps (such soaps promote the formation of hard, infrequent stools in formula-fed infants).^3,10^ In addition, oligofructose (OF), which is a nondigestible soluble dietary fiber, can be added to formula to increase the water-holding capacity of stool and decrease GI transit time, thereby promoting softer stools more similar to those of breastfed infants.^11-14^ Although a formula containing both increased *sn*-2 palmitate and OF (*sn*-2+OF) has been recently shown to improve stool consistency in double-blind, randomized, controlled, interventional trials (RCTs),^8,9^ the stool softening effectiveness of this formula has not been well characterized in a real-world setting. Previous RCTs^8,9^ on *sn*-2+OF formula were conducted in a highly controlled manner with close attention to study participants and frequent interaction with study physicians. It is not uncommon that efficacies of an intervention demonstrated in highly controlled RCTs may not always translate well into effectiveness outside of these controlled clinical settings.^15^ Additionally, a relatively large proportion of infants are fed with both breast milk and formula even at the first month of age (~50%),^16^ and this mixed feeding regimen, common in real-life settings, has not been studied in previous RCTs.^8,9^ Therefore, it is of clinical and research interest to characterize the stooling pattern and GI tolerance in infants who are mixed-fed.

This prospective study was conducted in a cohort of healthy Chinese infants receiving 1 of 3 different feeding regimens that had been independently chosen by parents prior to study start. The feeding regimens included infants exclusively breastfed, exclusively consuming *sn*-2+OF formula, or consuming both breast milk and the *sn*-2+OF formula (ie, mixed feeding). The primary objective was to characterize the level of hard stools and watery stools among infants of 3 feeding regimens in an observational, real-life setting. It is postulated that the incidences in infants fed the *sn*-2+OF formula would be low (≤2.5% for each) based on previously observed rates in interventional studies.^8,9^ The secondary objective was to compare the GI tolerance among infants of 3 feeding regimens utilizing the Infant Gastrointestinal Symptom Questionnaire (IGSQ).^17^

## Subjects and Methods

This was a 48-day, prospective, observational cohort study involving healthy term infants enrolled from the well-infant clinics of 24 hospitals in 14 major cities in China between September 2011 and April 2013. The study was conducted in accordance with the Declaration of Helsinki and Good Clinical Practices and was approved by the institutional ethics committees of all hospitals. Signed and dated written informed consent was obtained from all participants’ parents. Standardized training (eg, enrollment, data collection) was provided for all study investigators, and study monitors regularly examined the study implementation to ensure consistent study procedures and testing methods across different clinics.

### Participants

Healthy term infants were enrolled at approximately 42 days of age (35-49 days, date of birth = day 0), which coincided with the first normally scheduled well-infant clinic visit in China. Based on the reported feeding regimen during the 3 consecutive days prior to enrollment, and the decision by parent(s) prior to study entry to continue with this feeding regimen, infants were assigned into 1 of 3 groups: (1) exclusively breastfed infants, (2) formula-fed infants exclusively consuming a formula with *sn*-2+OF (infant formula enriched with α-lactalbumin and in which the fat blend contains 43% of the palmitate in the sn-2 position plus 3 g/L OF; ILLUMA, Wyeth Nutrition, Askeaton, Ireland), or (3) mixed-fed infants receiving both the *sn*-2+OF formula and breast milk in any proportion. Feeding regimens had been independently chosen by the parent(s) prior to study start, and formula was not provided to any parents. Each study site aimed to enroll infants approximately evenly into each of the 3 study groups, with recruitment monitored and temporarily suspended in a particular group as needed in order to maintain approximate balance. Infants were included in the study if their weight-for-age was ≥5th and ≤95th percentile at enrollment according to the World Health Organization growth standards.^18^ Screened infants were excluded if they (1) were consuming any type of formula other than the *sn*-2+OF formula at time of study enrollment, (2) had suspected or documented systemic or congenital conditions, (3) were receiving complementary foods or liquids (eg, more than ~5 mL of fruit/vegetable juice per day), and (4) were receiving any medication(s) or vitamin/mineral/herbal supplement(s) that were known or suspected to affect stool characteristics or any study outcomes.

### Study Visits and Outcome Measures

The study period was 48 days and included 3 clinic visits: baseline (study day 0; infant age ~42 days), study day 18 ± 3 (infant age ~60 days), and study day 48 ± 3 (infant age ~90 days). The study period and timing of visits were chosen to coincide with the typical schedule of well-infant clinic visits in China as well as to minimize the dropout rate and poor compliance that may be problematic in longitudinal studies. At each visit, infant anthropometric parameters (ie, weight, length, and head circumference) were measured using standardized techniques and calibrated equipment.^8,9^ The occurrence of adverse events (AEs) was also collected at these visits. Their severity and association with study feedings were assessed and determined by the study investigators. An AE was defined as any untoward, undesired, or unplanned event in the form of signs, symptoms, diseases, or laboratory or physiological observations occurring in an infant.^8,9^ In addition, phone calls were scheduled at study days 10 and 35 as well as at 2 weeks and 4 weeks after the last visit to collect AE information. As physician-reported GI AEs are informative on assessing GI tolerance to different feeding regimens, standard definitions for a subset of GI-related symptoms (eg, constipation and diarrhea) were provided to study investigators to ensure consistency in diagnosis.

To assess stool frequency and characteristics, parents were provided stool diaries and instructed to record the number of bowel movements and consistency of each stool passed by the infant over 3 consecutive days at home using a validated 5-point scale (1 = watery, 2 = runny, 3 = mushy soft, 4 = formed, or 5 = hard).^1,3^ These data were collected at 4 study intervals (study days 2-4, 15-17, 30-32, and 45-47). To assist with the assessment of stool consistency, the diaries included standardized photographs of stools corresponding to each point on the scale. The percentages of infants who had hard stools and watery stools were calculated for each study interval. For example, the percentage of infants who had hard stools at one study interval = (number of infants with at least 1 hard stool/[number of infants with complete 3-day data + number of infants with incomplete 3-day data but who had at least one hard stool]) × 100%. The percentage of infants who had watery stools was similarly calculated for each study interval. These calculations ensured that infants with a hard or watery stool were counted even if they did not provide a complete 3-day diary. Mean stool consistency score was also calculated for each study interval using the information recorded in the diaries.

A standardized, validated, and interviewer-assisted questionnaire, the IGSQ,^17^ was administered to parents at each study visit to assess GI tolerance of infants over the previous 7 days. The IGSQ consists of 21 items that allow parents to describe the frequency and intensity of infant GI signs and symptoms within 5 domains: stooling, vomiting, crying, fussiness, and flatulence. An overall index score was calculated from a subset of 13 items in the questionnaire as a measure of total GI symptom burden as previously described.^9,19^ The values for the IGSQ index score can range from 13 (low GI burden) to 65 (high GI burden). The IGSQ has been previously administered in 4 studies conducted in 3 countries (including China; total n = 836), and shown to be a valid and reliable method for assessment of infant GI-related behaviors.^17^

Parents were allowed to switch their infant to another feeding regimen at any time during the study, with continued follow-up dependent on the timing of the switch relative to study days 15 to 17 (when the second 3-day stool diary was completed). Infants who switched from one to another of the 3 study feeding regimens before this time point continued to participate in the study, while those who switched to a non–study regimen were withdrawn. Switches to a non–study regimen after study days 15 to 17 did not result in subject withdrawal.

### Statistical Analysis

The sample size target of 135 infants in the exclusively formula-fed group provided a probability ~70% of observing a rate for hard stools (or watery stools) ≤2.5% with an upper limit of the 90% 2-sided confidence interval (CI) ≤5.0%. The incidence rates were estimated based on previously published findings of 1.5% incidence rates of hard and watery stools each in a population fed the same formula^9^ while taking into consideration that the current study would be conducted in a less controlled environment. Assuming 10% attrition of the study population, approximately 145 infants needed to be enrolled. The sample sizes for the exclusively breastfed and mixed-fed groups were set at 145 each for consistency. An original sample size of 500 in the exclusively formula-fed group was planned to provide a probability >90% of observing a rate for hard stools (or watery stools) ≤2.5% with an upper limit of the 90% 2-sided CI ≤4.0%. However, slow subject recruitment limited the ability to achieve the originally determined sample size, and therefore the subject enrollment target was revised prior to data analysis.

Standard procedures (eg, Shapiro-Wilk tests, visual examinations of histograms, and box plots) were used to assess normality and variance homogeneity. Analysis of variance (ANOVA) and Fisher’s exact tests were used to compare the continuous and categorical variables, respectively, among the 3 groups at baseline (study day 0).

For the primary objective comparisons, 90% 2-sided CIs were calculated for the incidence rates of hard stools and watery stools using the Clopper-Pearson method^20^ for each time interval. Fisher’s exact tests were used to compare the incidence rates of hard and watery stools among the 3 groups at each time point. Due to the complications that could be introduced when subjects switched feeding regimens, 3 sensitivity analyses were done in order to evaluate the robustness of the results under various assumptions for the primary endpoints: (1) *pure analysis*: infants who switched feeding regimens during the stool data collection period or the day prior to the collection period were excluded from the analysis; (2) *24-hour rule analysis*: the day of the feeding regimen switch and the next day were assigned to the preswitch regimen and subsequent days were assigned to the postswitch regimen, with hard and watery stools assigned to the feeding group as indicated; and (3) *conservative analysis*: when the relevant feeding regimen was in doubt due to a switch, the event (hard or watery stool) was assigned to “formula-fed” if either of the regimens was formula-fed or to “mixed-fed” if the switch was between mixed-fed and breastfed.

For the secondary objective comparisons including stool consistency scores and IGSQ scores, ANOVA was performed to compare the 3 groups at each time point. Pairwise comparisons between each pair of means obtained from the overall ANOVA were also performed. Analyses were conducted using SAS (version 9.1.3, SAS Institute Inc, Cary, NC), and data were reported as mean/median and 90% CI unless otherwise noted.

## Results

### Study Population

**Figure 1.**
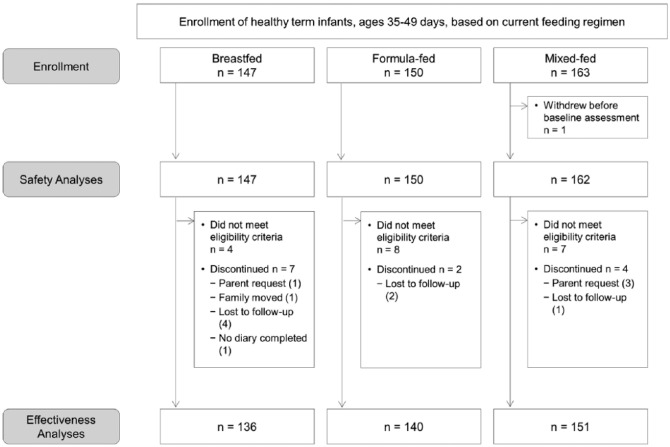
Enrollment and discontinuation of study participants. Breastfed, group of infants who were exclusively breastfed; formula-fed, group of infants who were exclusively fed with formula containing high *sn*-2-palmitate and oligofructose (*sn*-2+OF); mixed-fed, group of infants who were mixed fed with breast milk and *sn*-2+OF formula.

As shown in the study participant flow chart (Figure 1), a total of 460 infants were screened and enrolled from 24 sites, with each site enrolling between 2 and 39 infants. At 19 out of 24 sites, the enrolled infants were mostly evenly distributed across the 3 study groups, while at the remaining 5 sites, there was an uneven enrollment with one or more groups having no enrolled infants. One infant in the mixed-fed group withdrew before completion of the baseline assessment, and therefore was excluded from all subsequent analyses. All enrolled infants with baseline assessment data (n = 459) were included in the safety monitoring including anthropometric measures and AEs (safety population). Among 459 enrolled infants with baseline assessment data, 427 infants completed the study and were included for the effectiveness analysis (effectiveness population). The reasons for exclusion from the effectiveness population included the following: (1) 13 infants who did not complete the study; the main reasons for attrition included parent’s request for withdrawal (n = 4) and loss to follow-up (n = 7); and (2) 19 infants because their age at enrollment was outside of the inclusion criterion window.

Demographic and clinical characteristics of the infants in the effectiveness population were generally comparable among the 3 groups (Table 1). The majority of subjects (96%) were Han Chinese, 56% were male, 55% were delivered via cesarean section, and 0% attended day care. The formula-fed group had a slightly but significantly lower mean gestational age compared with the breastfed group (*P* = .004). Parental and household characteristics did not differ among the 3 groups (Table 1), except for mother’s education in years (*P* = .002), father’s education in years (*P* = .031), and father’s current smoking status (*P* = .02). Specifically, mothers and fathers of the formula-fed infants had significantly fewer years of education, and a larger proportion of fathers (46%) of formula-fed infants were current smokers. The feeding profile of the mixed-fed group was very stable over the study interval, with approximately 42 ± 19% of feeds per day provided by formula. The characteristics of the 459 infants in the safety population (data not shown) were similar to those of the 427 infants who completed the study.

**Table 1.** Baseline Demographic and Clinical Characteristics of the Study Population^a^.

	Breastfed (n = 136)	Formula-Fed (n = 140)	Mixed-Fed (n = 151)	Total (n = 427)	*P*
Infant characteristics					
Age at enrollment (days)	42.3 ± 3.7 (41.7-42.9)	42.2 ± 3.6 (41.5-42.8)	42.5 ± 3.4 (41.9-43.0)	42.3 ± 3.5 (42.0-42.6)	.75
Gestational age (weeks)	39.0 ± 1.0 (38.9-39.2)	38.6 ± 1.0 (38.5-38.8)	38.9 ± 1.0 (38.7-39.0)	38.9 ± 1.0 (38.8-38.9)	.004
Ethnicity, % Han	96	99	95	96	.20
Sex, % male	57.4	50.0	61.6	56.4	.13
Cesarean section delivery, %	49	59	56	55	.21
Infant attends daycare, %	0	0	0	0	1.0
Maternal characteristics					
Age (years)	29.2 ± 3.7 (28.6-29.8)	29.2 ± 3.8 (28.6-29.9)	29.9 ± 3.8 (29.3-30.5)	29.5 ± 3.8 (29.1-29.8)	.16
Marital status, % married	100	100	100	100	
Education (years)	15.8 ± 3.0 (15.3-16.3)	14.6 ± 2.8 (14.1-15.0)	15.3 ± 2.8 (14.9-15.8)	15.2 ± 2.9 (15.0-15.5)	.002
Occupation, % employed full-time	85.3	77.9	81.5	81.5	.12
Smoked during pregnancy, %	0.7	0.0	0.0	0.2	1.00
Current smoker, %	0.0	1.4	0.7	0.7	.53
Paternal characteristics					
Education (years)	15.6 ± 2.8 (15.1-16.0)	14.7 ± 3.0 (14.2-15.2)	15.4 ± 3.0 (14.9-15.8)	15.2 ± 2.9 (14.9-15.5)	.031
Occupation, % employed full-time	96.3	97.1	94.7	96.0	.28
Current smoker, %	29.4	45.7	37.1	37.5	.02

a Data presented as mean ± standard deviation (95% confidence interval) unless otherwise indicated. ANOVA and Fisher’s exact tests were used to compare the continuous and categorical variables, respectively, among the 3 groups. Breastfed, group of infants who were exclusively breastfed; formula-fed, group of infants who were exclusively fed with formula containing higher *sn*-2-palmitate and oligofructose (*sn*-2+OF); mixed-fed, group of infants who were mixed fed with breast milk and *sn*-2+OF formula.

Although feeding regimen changes were common prior to study enrollment, occurring in 167 of 460 screened infants (38%), few infants (n = 38; 8.6%) were switched to a different feeding regimen during the study period; switching occurred with higher frequency among infants in the mixed-fed versus in the formula-fed or breastfed groups. The cumulative number of feeding regimen switches was 31 times for 24 mixed-fed infants, which means 24 of the infants enrolled as mixed-fed changed their feeding regimen one or more times to breastfed or formula-fed at some time point during the study period. The cumulative number of regimen switches was 7 times for 7 formula-fed infants, and 7 times for 7 breastfed infants. The most common reasons for regimen changes included sufficient breast milk produced (for switching from mixed to exclusive breastfeeding) and insufficient breast milk produced (for switching from mixed to exclusive formula-feeding).

### Stool Consistency and Frequency

As stated in the statistical analysis section, 3 sensitivity analyses for the primary endpoints (incidences of hard stools and watery stools) were done (pure analysis, 24-hour rule analysis, and conservative analysis) to account for the potential complications that could be introduced when subjects switched feeding regimens. Due to the relatively low occurrence of feeding regimen switches, the results from the 3 sensitivity analyses were consistent with each other. Therefore, data from the pure analysis are presented below, and data from all 3 analyses are provided in Supplemental Table S1 (all supplemental tables are available in the online version of the journal).

#### Incidence of Hard Stools

The incidence (90% CI) of hard stools in the formula-fed group was 0.7% (0.0-3.3) throughout the study except at the visit corresponding to study days 15 to 17 (2.1% [0.6-5.3]; Table 2). Hard stool was not observed at any visit in the breastfed group and was only observed once in the mixed-fed group at study days 45 to 47 (Table 2). No statistically significant differences were observed among the 3 groups at any time point.

**Table 2.** Incidences of Hard and Watery Stools in the Effectiveness Population (Pure Analysis)^a,b^.

	Breastfed	Formula-Fed	Mixed-Fed
*Hard stool*			
Study days 2-4			
Proportion	0/135	1/140	0/147
Rate, % (90% CI)	0.0 (0.0, 2.2)	0.7 (0.0, 3.3)	0.0 (0.0, 2.0)
Study days 15-17			
Proportion	0/135	3/145	0/136
Rate, % (90% CI)	0.0 (0.0, 2.2)	2.1 (0.6, 5.3)	0.0 (0.0, 2.2)
Study days 30-32			
Proportion	0/138	1/141	0/137
Rate, % (90% CI)	0.0 (0.0, 2.2)	0.7 (0.0, 3.3)	0.0 (0.0, 2.2)
Study days 45-47			
Proportion	0/138	1/144	1/134
Rate, % (90% CI)	0.0 (0.0, 2.2)	0.7 (0.0, 3.3)	0.8 (0.0, 3.5)
*Watery stool*			
Study days 2-4			
Proportion	22/135	7/140	12/147
Rate, % (90% CI)	16.3 (11.3, 22.5)	5.0 (2.4, 9.2)^c^	8.2 (4.8, 12.9)^c^
Study days 15-17			
Proportion	17/135	4/145	10/136
Rate, % (90% CI)	12.6 (8.2, 18.3)	2.8 (1.0, 6.2)^c^	7.4 (4.0, 12.2)
Study days 30-32			
Proportion	10/138	6/141	10/137
Rate, % (90% CI)	7.3 (4.0, 12.0)	4.3 (1.9, 8.2)	7.3 (4.0, 12.1)
Study days 45-47			
Proportion	7/138	7/144	11/134
Rate, % (90% CI)	5.1 (2.4, 9.3)	4.9 (2.3, 8.9)	8.2 (4.7, 13.2)

a 90% confidence interval (CI) was computed using the Clopper-Pearson method.^20^

b Fisher’s exact tests were used to compare incidence rates of hard and watery stools among the 3 groups at each time point.

c Indicates statistically significant difference (*P* < .05) compared with the breastfed group. No other significant differences were observed. Breastfed, group of infants who were exclusively breastfed; formula-fed, group of infants who were exclusively fed with formula containing higher *sn*-2-palmitate and oligofructose (*sn*-2+OF); mixed-fed, group of infants who were mixed fed with breast milk and *sn*-2+OF formula.

#### Incidence of Watery Stools

The incidence of watery stools in the formula-fed group was ≤5.0% at all visits with the upper limit of the 90% CI ≤9.2% (Table 2). In contrast, the incidences of watery stools in the breastfed (5.1% to 16.3%) and mixed-fed groups (7.3% to 8.2%) were relatively high at all visits (Table 2). The formula-fed group had a significantly lower incidence of watery stools compared with the breastfed group at study days 2 to 4 (*P* = .003) and study days 15 to 17 (*P* = .002). The mixed-fed group also had a significantly lower incidence of watery stools compared with the breastfed group at study days 2 to 4 (*P* = .044). No additional significant differences were observed among the study groups.

#### Stool Consistency Score

**Figure 2.**
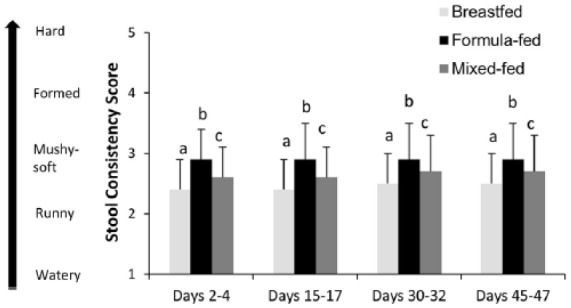
Stool consistency score for infants according to feeding groups at time points of evaluation. Mean ± SD stool consistency score of infants at each study visit. ANOVA followed by pairwise comparisons was conducted to compare groups at each time points. Bars with different letters (a, b, c) are significantly different (*P* < .05). Breastfed, group of infants who were exclusively breastfed; formula-fed, group of infants who were exclusively fed with formula containing higher *sn*-2-palmitate and oligofructose (*sn*-2+OF); mixed-fed, group of infants who were mixed fed with breast milk and *sn*-2+OF formula.

The mean stool consistency score differed significantly among the 3 study groups (overall *P* < .001; Figure 2) in a similar pattern throughout the study: the formula-fed group had the highest mean scores, followed by the mixed-fed group, and the breastfed group had the lowest mean scores. The mean scores for all groups were between 2 (runny) and 3 (mushy-soft), and the magnitude of the differences was small (eg, mean ± SD scores of formula-fed, mixed-fed, and breastfed were 2.9 ± 0.6, 2.7 ± 0.5, and 2.5 ± 0.5 at study end days 45-47, overall *P* < .001).

#### Stool Frequency

Mean daily stool frequency differed significantly among feeding groups at each assessment interval (all overall and pairwise *P* < .001). The formula-fed group had the fewest number of stools per day (1.8 ± 1.3 and 1.3 ± 0.9 at study days 2-4 and 45-47, respectively), followed by the mixed-fed group (2.7 ± 1.9 and 1.9 ± 1.6, respectively), while the breastfed group had the most stools per day (3.9 ± 2.1 and 2.5 ± 1.4, respectively).

### GI Tolerance

**Figure 3.**
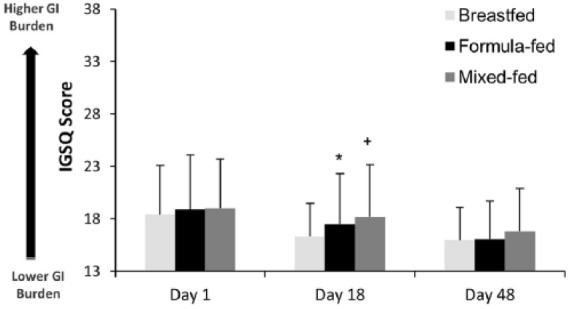
Gastrointestinal tolerance score for infants according to feeding groups at time points of evaluation. Mean ± SD IGSQ score of infants at each study visit. ANOVA followed by pairwise comparisons were conducted to compare groups at each time points. *P = .03 and ^+^*P* = .0003 indicate significant differences from breastfed group. Breastfed, group of infants who were exclusively breastfed; formula-fed, group of infants who were exclusively fed with formula containing higher *sn*-2-palmitate and oligofructose (*sn*-2+OF); mixed-fed, group of infants who were mixed fed with breast milk and *sn*-2+OF formula; IGSQ, Infant Gastrointestinal Symptom Questionnaire.

IGSQ scores, an indicator of GI tolerance (the lowest score possible is 13 and the highest score is 65, with lower scores representing better tolerance), were relatively low for all 3 groups throughout the study, indicating good GI tolerance for all feeding regimens (Figure 3). No differences in IGSQ scores among the 3 study groups were detected at study days 1 (overall *P* = .51) or 48 (overall *P* = .12). IGSQ scores (mean ± SD) at day 18 differed among the 3 groups: the formula-fed (17.5 ± 4.8, *P* = .03) and mixed-fed groups (18.2 ± 5.0, *P* = .0003) had higher scores than the breastfed group (16.3 ± 3.2), while no significant difference (*P* = .15) between the formula-fed and mixed-fed groups was observed.

### Anthropometric Measures and Adverse Events

We collected anthropometric parameters (eg, weight, length, and head circumference) and comprehensive AE information for all 459 infants who completed the baseline assessment. Throughout the study period, infants in all 3 groups grew comparably. Although statistical analysis of the anthropometry data was not performed, infant length, weight, and head circumference was very similar among the 3 groups, and the small numeric differences did not appear to be clinically relevant (Supplemental Table S2).

The majority of infants (81.5%) who participated in the study did not manifest any AEs (Supplemental Table S3). The percentages of subjects with any AEs in the breastfed, formula-fed, and mixed-fed groups throughout the entire study period (~48 days) plus a 4-week poststudy follow-up after the last clinical visit were 22%, 16%, and 18%, respectively. Notably, the physician-reported constipation and diarrhea incidences were very low for all 3 study groups (0% and 3.4% for breastfed group, 1.3% and 1.3% for formula-fed group, and 1.9% and 1.9% for mixed-fed groups, respectively), which are consistent with the parent-reported low incidence rates of the hard and watery stools observed in the study. A total of 4 serious AEs (resulting in hospitalization) were reported: one report of bronchopneumonia in the formula-fed group, one report of bronchopneumonia in the mixed-fed group, as well as one report of bronchopneumonia and one report of pneumonia in the breastfed group. Only one AE led to study discontinuation (in the formula-fed group; due to umbilical hernia). None of the serious AEs were considered related to infants’ feeding.

## Discussion

Formula-fed infants often have stools that are harder than those of breast-fed infants, as demonstrated in previous studies.^1,3,4,21^ However, most of these studies reported group means for stool consistency and did not focus on specific incidence of either hard or watery stools, which are primary concerns of parents. In addition, data describing the characteristics of stool consistency and GI tolerance in association with different feeding regimens in healthy Chinese infants in a real-life, observational setting are scarce.

Our results demonstrate that (1) infants from all 3 groups (exclusively breastfed, exclusively *sn*-2+OF formula fed, as well as mixed fed of breast milk and the *sn*-2+OF formula) had similarly low incidences of hard stools including the infants fed exclusively with the *sn*-2+OF formula; (2) incidence of watery stools was consistently lower in the group exclusively receiving formula than in the other groups; and (3) IGSQ scores were low (indicating good tolerance) in all groups. The only significant difference in IGSQ occurred at day 18 of the study at which time the breastfed group had a slightly lower IGSQ score than the other groups. Stooling frequency differed between groups at all study intervals, but the differences were small and generally consistent with those reported elsewhere.^22^ It is also notable that only a few switches of feeding regimen were observed in the study population, especially in infants exclusively fed with *sn*-2+OF formula or breast milk, despite literature^1,2^ suggesting that feeding regimen switching is common at this age.

Multiple compositional differences between breast milk and traditional infant formula likely contribute to the observation that formula-fed infants experience more hard stools compared with breastfed infants. Breast milk is rich in triglycerides with ~66% of its palmitic acid in the *sn*-2 position.^23^ In contrast, the palmitate of traditional formula fat blends is predominantly (>80%) located in the *sn*-1 and *sn*-3 positions.^24^ Additionally, breast milk contains complex carbohydrates (prebiotic fibers)^25^ that are not digestible in the small intestine and can arrive intact in the colon and produce softer stools. In the past decade, fat blends with increased levels of *sn*-2 palmitate and prebiotic fibers such as OF became available and have been demonstrated to assume some similar functions when added to infant formula. Specifically, metabolic balance studies have demonstrated that infants fed formulas containing high *sn*-2 palmitate have better calcium and fatty acid absorption than infants receiving conventional formulas.^26^ A number of studies have demonstrated that infants fed formulas containing high *sn*-2 palmitate have reduced levels of stool calcium-palmitate soaps.^8,9,27^ Some^8,9,27^ but not all^28^ studies have demonstrated that infants fed formulas featuring high *sn*-2 palmitate have softer stools than infants fed traditional formulas. OF serves as a substrate for specific beneficial colonic bacteria, such as bifidobacteria,^29^ and increases stool bulk.^30^ Infants receiving formula supplemented with OF have softer stools than infants receiving control formulas.^11,14,31^ Formula supplemented with OF and inulin also produced softer stools than a control formula at some, but not all, time points in a 4-month study.^13^ Formula supplemented with both OF and *sn*-2 palmitate has been studied in previous RCTs,^8,9^ and the combination of OF and high *sn*-2 palmitate resulted in softer stools than formula containing the structured lipid alone.^8^ To the best of our knowledge, the current study is the first one to evaluate the effectiveness of a *sn*-2+OF formula in a real-life setting. The findings of the present study have further confirmed that infants fed formulas featuring *sn*-2+OF have low incidence of hard stools.

Concerns have been raised that formulas containing prebiotics produce softer stools and such stool softening may proceed to an extreme, which could result in excessive fluid loss and dehydration.^32^ Numerous lines of evidence produce data to the contrary. Closa-Monasterolo et al^13^ evaluated hydration status in infants consuming a formula containing both inulin and OF utilizing both urine electrolyte concentrations and serum parameters (electrolytes and urea concentrations); all parameters were within normal ranges. Wernimont et al^31^ utilized a physician rating scale including mucous membranes, skin turgor, and other parameters to determine hydration status of infants receiving a formula supplemented with OF compared with a control formula or breast milk; all infants had normal hydration status. Feeding the combination of OF and high *sn*-2 palmitate resulted in normal hydration status as determined by urine osmolality and specific gravity.^8^ In the current study, the infants who consumed *sn*-2+OF formula exclusively had comparably low or even lower incidence rates of watery stools than either the breastfed or mixed-fed groups. Although the current study did not evaluate the infants’ hydration status, the low incidence of watery stools and the lack of dehydration-related AEs among infants receiving formula containing *sn*-2+OF, coupled with more specific hydration-related data from previous intervention studies, clearly demonstrate the safety of additions of both *sn*-2 palmitate and OF (43% *sn*-2 palmitate fat blend plus 3 g/L OF) to infant formula.

Parents pay a great amount of attention to the GI tolerance of their infants and the acceptance of the feeding regimen being used.^21^ GI tolerance is often evaluated by a general consideration of stool consistency, spitting-up, colic, general intolerance, and failure to thrive, with infant formulas generally perceived to be more poorly tolerated than breast milk.^4,21^ Riley et al^17^ developed a parent-reported tool, the IGSQ, to evaluate GI tolerance in infants. The IGSQ has been validated using data from 3 countries (the United States, China, and the Philippines).^17^ A comparison of infants who were either breastfed or receiving traditional infant formula (both groups composed of healthy infants with low GI distress) found a 2-point difference between groups; the primary rating differences were a slightly higher frequency of hard stools, fussiness, and spitting-up in formula-fed infants.^17^ Specific formula characteristics, such as enrichment of high *sn*-2 palmitate and OF, have been demonstrated to improve the GI tolerance in clinical studies.^8,9,31^ The data from the current study demonstrated that each feeding regimen (breastfed, *sn*-2+OF formula, or mixed-feeding of breast milk and *sn*-2+OF formula) in a real-life setting was well tolerated and differences between infants receiving breast milk and the formula were marginal. At day 18, the IGSQ for both the formula alone and the mixed groups was significantly higher than the breastfed group (mean score difference 1.2 and 1.9, respectively), although at the time points before (day 1) and after (study end day 48) the scores for all 3 groups were similar. These small differences would require only 1 or 2 items on the IGSQ to be rated above a score of 1 when compared with the breastfed group. Previous randomized, blinded studies that employed the IGSQ also found that feeding *sn*-2+OF formulas resulted in low IGSQ scores that were not significantly different from breastfed infants.^8,9^ Additionally, only a few feeding regimen switches in *sn*-2+OF formula group were observed in the study population, which supports that the studied formula was well tolerated.

The present study findings have high generalizability in healthy Chinese infants. Study participants were recruited from 24 sites in 14 major cities, providing a wide representation among different regions of China. Additionally, the current study included a group of infants receiving mixed feedings. Most studies avoid consideration of infants simultaneously receiving both breast milk and formula, although mothers often introduce formula feeding as they prepare to wean or as maternity leave ends and they return to work. The findings on the mixed-fed group contribute to our knowledge on the stool characteristics and GI tolerance of this relatively understudied population. Furthermore, infants were allowed to switch feeding regimens to reflect the real-life setting, and the effect of switching on the primary outcomes in our analysis was carefully considered by employing 3 analysis modes: pure, conservative, and 24-hour rule analysis.

The study was carefully designed to avoid selection bias by choosing representative study sites and providing rigorous training to research staff and close monitoring of the study implementation. Since we enrolled infants who had already been fed the *sn*-2+OF formula for at least 3 days prior to enrollment, there may be a possibility that the study did not capture the infants who had not tolerated the *sn*-2+OF formula when it was first consumed and already switched to another type of formula. Therefore, the study may have underestimated the feeding intolerance of the *sn*-2+OF formula due to this possible selection bias. However, we expect minimal selection bias because in the previous 2 RCTs of the *sn*-2+OF formula,^8,9^ none of 131 infants withdrew from the *sn*-2+OF formula group due to feeding intolerance, indicating the formula was well tolerated when it was first consumed. In addition, the stool consistency data (eg, incidence rate of hard and watery stools) relied only on parental reports, and thus may be more prone to reporting biases compared with more objective methods; nonetheless, it is unlikely that parents especially in the formula-fed group would underreport undesirable incidences. Detailed instructions (including photographic examples of stools corresponding to each point on the scale) were provided to parents to facilitate completion of the stool diaries in a standardized manner. We also recognize that inclusion of infants fed other formulas would allow more direct comparison of our results with those of RCTs that have included control formulas; however, infants consuming non–study formulas were not enrolled in the present study. In addition, the homogeneous study population (96% Han Chinese) may limit generalizability to other groups. Future studies should consider including infants fed other formulas and from more varied racial and ethnic backgrounds, and implementing more direct measures, that is, collecting stool samples and quantifying stool palmitate soaps, total soaps, calcium, short-chain fatty acids, and gut microbiota, in order to better quantify the potential GI benefits of *sn*-2+OF formula.

## Conclusions

The present study demonstrates that neither hard stools nor watery stools is a common occurrence in healthy Chinese infants fed a formula containing high *sn*-2 palmitate and OF. Stool consistency scores were close to 3 (mushy-soft) for all feeding regimens at all visits; nonetheless, the scores were slightly higher in the formula-fed group versus either the breastfed or mixed-fed groups. Additionally, the *sn*-2+OF formula was well tolerated based on both parent questionnaire (IGSQ) and physician-reported GI study events, as well as a low feeding regimen switching rate due to undesirable GI symptoms. These results in an observational, real-life setting are consistent with data from previous RCTs showing the beneficial effects of term infant formula containing *sn*-2+OF on GI outcomes in formula-fed infants.

## Supplementary Material

Supplementary material

Supplementary material

Supplementary material
