# Supplementary material for: Infant Feeding Regimens and Gastrointestinal Tolerance: A Multicenter, Prospective, Observational Cohort Study in China
Source: Glob Pediatr Health. 2018 Jan 9;5:2333794X17750271. doi: 10.1177/2333794X17750271 (PMC5764142; doi:10.1177/2333794X17750271)
Supplement: Supplementary material [file S1_Table.docx]

**S1 Table.** Incidences of hard and watery stools in the effectiveness population (sensitivity analyses).

|  | **Pure Analysis** | | | **24-Hour Rule Analysis** | | | **Conservative Analysis** | | |
| --- | --- | --- | --- | --- | --- | --- | --- | --- | --- |
|  | **Breastfed** | **Formula-fed** | **Mixed-fed** | **Breastfed** | **Formula-fed** | **Mixed-fed** | **Breastfed** | **Formula-fed** | **Mixed-fed** |
| **Hard Stool** |  |  |  |  |  |  |  |  |  |
| *Study Days 2-4* |  |  |  |  |  |  |  |  |  |
| Proportion | 0 / 135 | 1 / 140 | 0 / 147 | 0 / 135 | 1 / 140 | 0 / 147 | 0 / 135 | 1 / 140 | 0 / 147 |
| Rate, %  (90% CI ^1^) | 0.0  (0.0, 2.2) | 0.7  (0.0, 3.3) | 0.0  (0.0, 2.0) | 0.0  (0.0, 2.2) | 0.7  (0.0, 3.3) | 0.0  (0.0, 2.0) | 0.0  (0.0, 2.2) | 0.7  (0.0, 3.3) | 0.0  (0.0, 2.0) |
| *Study Days 15-17* |  |  |  |  |  |  |  |  |  |
| Proportion | 0 / 135 | 3 / 145 | 0 / 136 | 0 / 135 | 3 / 146 | 0 / 136 | 0 / 135 | 3 / 146 | 0 / 136 |
| Rate, %  (90% CI) | 0.0  (0.0, 2.2) | 2.1  (0.6, 5.3) | 0.0  (0.0, 2.2) | 0.0  (0.0, 2.2) | 2.1  (0.6, 5.2) | 0.0  (0.0, 2.2) | 0.0  (0.0, 2.2) | 2.1  (0.6, 5.2) | 0.0  (0.0, 2.2) |
| *Study Days 30-32* |  |  |  |  |  |  |  |  |  |
| Proportion | 0 / 138 | 1 / 141 | 0 / 137 | 0 / 139 | 2 / 142 | 0 / 137 | 0 / 139 | 2 / 142 | 0 / 137 |
| Rate, %  (90% CI) | 0.0  (0.0, 2.2) | 0.7  (0.0, 3.3) | 0.0  (0.0, 2.2) | 0.0  (0.0, 2.1) | 1.4  (0.3, 4.4) | 0.0  (0.0, 2.2) | 0.0  (0.0, 2.1) | 1.4  (0.3, 4.4) | 0.0  (0.0, 2.2) |
| *Study Days 45-47* |  |  |  |  |  |  |  |  |  |
| Proportion | 0 / 138 | 1 / 144 | 1 / 134 | 0 / 138 | 1 / 144 | 1 / 134 | 0 / 138 | 1 / 144 | 1 / 134 |
| Rate, %  (90% CI) | 0.0  (0.0, 2.2) | 0.7  (0.0, 3.3) | 0.8  (0.0, 3.5) | 0.0  (0.0, 2.2) | 0.7  (0.0, 3.3) | 0.8  (0.0, 3.5) | 0.0  (0.0, 2.2) | 0.7  (0.0, 3.3) | 0.8  (0.0, 3.5) |
| **Watery Stool** |  |  |  |  |  |  |  |  |  |
| *Study Days 2-4* |  |  |  |  |  |  |  |  |  |
| Proportion | 22 / 135 | 7 / 140 | 12 / 147 | 22 / 135 | 7 / 140 | 12 / 147 | 22 / 135 | 7 / 140 | 12 / 147 |
| Rate, %  (90% CI) | 16.3  (11.3, 22.5) | 5.0  (2.4, 9.2) | 8.2  (4.8, 12.9) | 16.3  (11.3, 22.5) | 5.0  (2.4, 9.2) | 8.2  (4.8, 12.9 ) | 16.3  (11.3, 22.5) | 5.0  (2.4, 9.2) | 8.2  (4.8, 12.9) |
| *Study Days 15-17* |  |  |  |  |  |  |  |  |  |
| Proportion | 17 / 135 | 4 / 145 | 10 / 136 | 17 / 135 | 4 / 146 | 10 / 136 | 17 / 135 | 4 / 146 | 10 / 136 |
| Rate, %  (90% CI) | 12.6  (8.2, 18.3) | 2.8  (1.0, 6.2) | 7.4  (4.0, 12.2) | 12.6  (8.2, 18.3) | 2.7  (0.9, 6.2) | 7.4  (4.0, 12.2) | 12.6  (8.2, 18.3) | 2.7  (0.9, 6.2) | 7.4  (4.0, 12.2) |
| *Study Days 30-32* |  |  |  |  |  |  |  |  |  |
| Proportion | 10 / 138 | 6 / 141 | 10 / 137 | 10 / 139 | 6 / 142 | 10 / 137 | 10 / 139 | 6 / 142 | 10 / 137 |
| Rate, %  (90% CI) | 7.3  (4.0, 12.0) | 4.3  (1.9, 8.2) | 7.3  (4.0, 12.1) | 7.2  (4.0, 11.9) | 4.2  (1.9, 8.2) | 7.3  (4.0, 12.1) | 7.2  (4.0, 11.9) | 4.2  (1.9, 8.2) | 7.3  (4.0, 12.1) |
| *Study Days 45-47* |  |  |  |  |  |  |  |  |  |
| Proportion | 7 / 138 | 7 / 144 | 11 / 134 | 7 / 138 | 7 / 144 | 11 / 134 | 7 / 138 | 7 / 144 | 11 / 134 |
| Rate, %  (90% CI) | 5.1  (2.4, 9.3) | 4.9  (2.3, 8.9) | 8.2  (4.7, 13.2) | 5.1  (2.4, 9.3) | 4.9  (2.3, 8.9) | 8.2  (4.7, 13.2) | 5.1  (2.4, 9.3) | 4.9  (2.3, 8.9) | 8.2  (4.7, 13.2) |
