# Supplementary material for: Infant Feeding Regimens and Gastrointestinal Tolerance: A Multicenter, Prospective, Observational Cohort Study in China
Source: Glob Pediatr Health. 2018 Jan 9;5:2333794X17750271. doi: 10.1177/2333794X17750271 (PMC5764142; doi:10.1177/2333794X17750271)
Supplement: Supplementary material [file S2_Table.docx]

**S2 Table.** Infant anthropometric measures in safety population.^1,2^

|  | **Breastfed** | **Formula-fed** | **Mixed-fed** |
| --- | --- | --- | --- |
| ***Length (cm)*** |  |  |  |
| Visit 1 | 56.1 ± 2.3 (n = 137) | 55.4 ± 3.0 (n = 148) | 56.0 ± 2.7 (n = 155) |
| Visit 2 | 58.7 ± 2.7 (n = 133) | 57.9 ± 3.1 (n = 142) | 58.8 ± 2.9 (n = 138) |
| Visit 3 | 61.4 ± 3.0 (n = 129) | 60.9 ± 3.5 (n = 136) | 61.7 ± 3.2 (n = 130) |
| ***Weight (kg)*** |  |  |  |
| Visit 1 | 5.0 ± 0.5 (n = 137) | 4.9 ± 0.5 (n = 148) | 5.0 ± 0.5 (n = 155) |
| Visit 2 | 5.8 ± 0.6 (n = 133) | 5.6 ± 0.5 (n = 142) | 5.8 ± 0.6 (n = 138) |
| Visit 3 | 6.7 ± 0.7 (n = 129) | 6.5 ± 0.6 (n = 137) | 6.6 ± 0.7 (n = 130) |
| ***Head Circumference (cm)*** |  |  |  |
| Visit 1 | 37.9 ± 1.4 (n = 137) | 37.6 ± 1.7 (n = 147) | 37.9 ± 1.3 (n = 152) |
| Visit 2 | 39.1 ± 1.4 (n = 133) | 38.8 ± 1.6 (n = 141) | 39.3 ± 1.5 (n = 134) |
| Visit 3 | 40.6 ± 1.5 (n = 129) | 40.0 ± 1.6 (n = 135) | 40.6 ± 1.6 (n = 126) |
