# Supplementary material for: Infant Feeding Regimens and Gastrointestinal Tolerance: A Multicenter, Prospective, Observational Cohort Study in China
Source: Glob Pediatr Health. 2018 Jan 9;5:2333794X17750271. doi: 10.1177/2333794X17750271 (PMC5764142; doi:10.1177/2333794X17750271)
Supplement: Supplementary material [file S3_Table.docx]

**S3 Table.** Adverse events from enrollment through 4 weeks after the last clinical visit.

|  | **Breastfed  (n = 147)** | **Formula-fed  (n = 150)** | **Mixed-fed  (n = 162)** | **Total  (n = 459)** |
| --- | --- | --- | --- | --- |
| Total number (%) of subjects with adverse event(s) | 32 (21.8) | 24 (16.0) | 29 (17.9) | 85 (18.5) |
| *Gastrointestinal disorders* | *9 (6.1)* | *9 (6.0)* | *8 (4.9)* | *26 (5.7)* |
| Constipation | 0 (0.0) | 2 (1.3) | 3 (1.9) | 5 (1.1) |
| Diarrhea | 5 (3.4) | 2 (1.3) | 3 (1.9) | 10 (2.2) |
| Dyspepsia | 3 (2.0) | 1 (0.7) | 2 (1.2) | 6 (1.3) |
| Enteritis | 1 (0.7) | 0 (0.0) | 1 (0.6) | 2 (0.4) |
| Flatulence | 0 (0.0) | 1 (0.7) | 1 (0.6) | 2 (0.4) |
| Gastrointestinal pain | 0 (0.0) | 1 (0.7) | 0 (0.0) | 1 (0.2) |
| Gastroesophageal reflux disease | 0 (0.0) | 1 (0.7) | 0 (0.0) | 1 (0.2) |
| Infantile spitting up | 1 (0.7) | 0 (0.0) | 0 (0.0) | 1 (0.2) |
| Umbilical hernia | 0 (0.0) | 1 (0.7) | 0 (0.0) | 1 (0.2) |
| *General disorders and administration site conditions* | *2 (1.4)* | *1 (0.7)* | *1 (0.6)* | *4 (0.9)* |
| Crying | 1 (0.7) | 1 (0.7) | 0 (0.0) | 2 (0.4) |
| Pyrexia | 1 (0.7) | 0 (0.0) | 1 (0.6) | 2 (0.4) |
| *Infections and infestations* | *15 (10.2)* | *15 (10.0)* | *17 (10.5)* | *47 (10.2)* |
| Bronchiolitis | 0 (0.0) | 1 (0.7) | 0 (0.0) | 1 (0.2) |
| Bronchitis | 2 (1.4) | 0 (0.0) | 2 (1.2) | 4 (0.9) |
| Bronchopneumonia | 1 (0.7) | 1 (0.7) | 1 (0.6) | 3 (0.7) |
| Candidiasis | 0 (0.0) | 1 (0.7) | 0 (0.0) | 1 (0.2) |
| Dacryocystitis | 0 (0.0) | 1 (0.7) | 0 (0.0) | 1 (0.2) |
| Nasopharyngitis | 1 (0.7) | 1 (0.7) | 0 (0.0) | 2 (0.4) |
| Pharyngitis | 1 (0.7) | 0 (0.0) | 0 (0.0) | 1 (0.2) |
| Pneumonia | 1 (0.7) | 0 (0.0) | 0 (0.0) | 1 (0.2) |
| Respiratory tract infection | 0 (0.0) | 1 (0.7) | 1 (0.6) | 2 (0.4) |
| Upper respiratory tract infection | 10 (6.8) | 11 (7.3) | 13 (8.0) | 34 (7.4) |
| *Injury, poisoning and procedural complications* | *0 (0.0)* | *0 (0.0)* | *1 (0.6)* | *1 (0.2)* |
| Fall | 0 (0.0) | 0 (0.0) | 1 (0.6) | 1 (0.2) |
| *Metabolism and nutrition disorders* | *0 (0.0)* | *1 (0.7)* | *1 (0.6)* | *2 (0.4)* |
| Decreased appetite | 0 (0.0) | 1 (0.7) | 0 (0.0) | 1 (0.2) |
| Vitamin D deficiency | 0 (0.0) | 0 (0.0) | 1 (0.6) | 1 (0.2) |
| *Pregnancy, puerperium and perinatal conditions* | *1 (0.7)* | *1 (0.7)* | *0 (0.0)* | *2 (0.4)* |
| Jaundice neonatal | 1 (0.7) | 0 (0.0) | 0 (0.0) | 1 (0.2) |
| Neonatal disorder | 0 (0.0) | 1 (0.7) | 0 (0.0) | 1 (0.2) |
| *Respiratory, thoracic and mediastinal disorders* | *1 (0.7)* | *1 (0.7)* | *1 (0.6)* | *3 (0.7)* |
| Cough | 1 (0.7) | 0 (0.0) | 1 (0.6) | 2 (0.4) |
| Nasal obstruction | 0 (0.0) | 1 (0.7) | 0 (0.0) | 1 (0.2) |
| *Skin and subcutaneous tissue disorders* | *8 (5.4)* | *3 (2.0)* | *4 (2.5)* | *15 (3.3)* |
| Eczema | 8 (5.4) | 2 (1.3) | 4 (2.5) | 14 (3.1) |
| Skin erosion | 0 (0.0) | 1 (0.7) | 0 (0.0) | 1 (0.2) |
